# Evaluating the Prognostic Significance of Circulating Biomarkers of End Organ Damage in Hypertension

**DOI:** 10.3390/jcm14175935

**Published:** 2025-08-22

**Authors:** Elliot Mbeta, Katie Williams, James Yates, Rajiv Sankaranarayanan, Peter Penson, Gregory Y. H. Lip, Garry McDowell

**Affiliations:** 1 Liverpool Centre for Cardiovascular Science at Liverpool John Moores University (LJMU), University of Liverpool and Liverpool Heart and Chest Hospital, Liverpool L8 8TX, UK; e.mbeta@2023.ljmu.ac.uk (E.M.); k.a.williams@2023.ljmu.ac.uk (K.W.); j.yates@2023.ljmu.ac.uk (J.Y.); lipgy@liverpool.ac.uk (G.Y.H.L.); 2 School of Pharmacy and Biomolecular Sciences, Liverpool John Moores University, Liverpool L3 3AF, UK; 3 Department of Cardiology, Aintree University Hospital, University Hospitals of Liverpool Group, Liverpool L9 7AL, UK; 4 Danish Centre for Health Services Research, Department of Clinical Medicine, Aalborg University, 9220 Aalborg East, Denmark; 5 Cardiovascular and Metabolic Medicine, Institute of Life Course and Medical Sciences, University of Liverpool, Liverpool L8 8TX, UK; 6 Research Laboratory, Liverpool Heart and Chest Hospital, Liverpool L14 3PE, UK

**Keywords:** biomarkers, hypertension, heart failure, major adverse cardiovascular events, end organ damage

## Abstract

**Background:** Most patients with hypertension exhibit elevated and detectable levels of natriuretic peptides, particularly BNP and NT-proBNP, as well as troponin concentrations. However, the prognostic relevance of this finding has not been clearly established in patients who have hypertension without heart failure (HF). In this review, we aimed to evaluate the prognostic utility of BNP/NT-proBNP alongside troponin T/I for risk stratification in hypertensive patients, excluding those with HF. **Methods:** This systematic review was registered in PROSPERO (CRD42024552031). A systematic literature search was conducted using two online databases, Ovid Medline and Web of Science, to identify studies. Data retrieved from articles were used in line with the PRISMA statement guidelines. Participants were aged ≥ 18 years with hypertension. The primary end point was a major adverse cardiac event (MACE) and its individual components. Descriptive synthesis was performed, and data are presented in tabular form. **Results:** Seventeen studies (70,021 participants) were retrieved for analysis comprising eight prospective cohort studies, six randomized controlled trials, and three retrospective studies. The review evaluated cardiac biomarkers: BNP (*n* = 6), NT proBNP (n=9), troponin T (*n* = 4), and troponin I (*n* = 7). Studies predicted composite MACE (*n* = 8), all-cause mortality (*n* = 7), HF (*n* = 6), and atrial fibrillation (*n* = 3) outcomes. Cardiac biomarkers showed a strong association with reported outcomes. However, heterogeneity in biomarker thresholds and methodologies limited comparability. **Conclusions:** The obtained results suggest that elevated cardiac biomarkers BNP/NT-proBNP and troponin I are associated with significantly higher risk of MACE and are powerful predictors in clinical setting. However, large-scale studies are required to validate the robustness and prognostic utility of these biomarkers

## 1. Introduction

Hypertension is a major risk factor for heart failure (HF) [1,2], atrial fibrillation (AF) [3], stroke [4], and other cardiovascular diseases. The World Health Organization (WHO) estimates that 1.3 billion adults aged 30–79 years are hypertensive [5]. Furthermore, the WHO reports that approximately 46% of hypertensive patients are unaware they have the condition, and acknowledges that only 42% are diagnosed and treated. Major disparities in hypertension risk, awareness, and management have been reported based on the country’s socioeconomic level [6,7]. In 2019, approximately 1 billion hypertensive patients lived in low- and middle-income regions [8] and cardiovascular diseases (CVD) were the leading causes of death worldwide, which, in addition, resulted in significant morbidity and healthcare costs [9,10].

Several risk factors are associated with the development of hypertension through a complex interaction of genetic and lifestyle behaviours. Factors such as excessive salt, fat, and harmful alcohol consumption, physical inactivity, and poor management of stress increase the risk of hypertension [5,11]. The association between hypertension and progression to heart failure and other cardiac events has collectively imposed an enormous economic burden [12] thus requiring new strategies for early risk stratification.

Risk stratification is the cornerstone of cardiovascular disease (CVD) prevention and management, particularly for hypertensive patients at risk for major adverse cardiovascular events (MACE). While current clinical guidelines recommend tools such as QRISK3 [13] and SCORE2 [14] to assess cardiovascular risk based on conventional risk factors, emerging evidence suggests these tools may not adequately capture the full complexity of cardiovascular diseases [15,16,17]. Thus, incorporation of cardiac biomarkers of myocardial stress or injury have been suggested to provide additional prognostic value in cardiovascular risk stratification.

Circulating plasma biomarkers, particularly natriuretic peptides (NPs) and cardiac troponins, are indicators of biological processes [18]. Currently, B-type natriuretic peptide (BNP) and N-terminal pro-B-type natriuretic peptide (NTproBNP)), in addition to cardiac troponins, are well-established markers for the diagnosis of heart failure and acute coronary syndromes, respectively [19,20], and have been incorporated into numerous international clinical guidelines [21,22]. However, the utility of NP and cardiac troponins in clinical practice for risk stratification remains underutilized in hypertensive patients who are at risk of developing HF and other cardiac events

To our best knowledge, no comprehensive overview of the evidence for the prognostic value of cardiac biomarkers, especially BNP/NT-proBNP and troponin T and I, on major adverse cardiac events (MACE) in hypertension has been reported, despite the widely accepted and growing interest in the use of circulating plasma biomarkers for risk stratification. The aim of this systematic review was to determine the prognostic value of BNP/NT-proBNP and troponin T and I on major adverse cardiac events in patients with hypertension without heart failure.

## 2. Materials and Methods

This systematic review followed PRISMA statement guidelines for reporting systematic reviews [23,24]. The predefined study protocol for this systematic review was registered at the International Prospective Register of Systematic Reviews (PROSPERO) [25] (No. CRD42024552031). The PICOT framework for the systematic review is as follows (Table 1):

### 2.1. Database Searches and Inclusion Criteria

A systematic literature search was conducted by two reviewers (EM and JY) in two online databases: Ovid Medline and Web of Science, covering studies published within a 10-year period from 2013 to 2024, with the final search completed on 20 February 2025. The included studies met the following eligibility criteria: (1) adults > 18 years with diagnosis of hypertension, (2) cardiac biomarkers (BNP or NT- proBNP, troponin I or troponin T) reported, (3) reported primary or secondary outcomes, (4) eligible study designs included randomized controlled trials, cohort studies, case-control studies, and cross-sectional studies. To ensure relevance and data availability, filters were applied to include only original research articles with accessible abstracts containing sufficient data for extraction. Studies not reporting outcomes as hazard ratios or odds ratios were excluded alongside studies conducted in vitro. Furthermore, studies in which hypertension was not the primary focus but merely included as a covariate were excluded from the analysis. Conference abstracts, dissertations, case reports, feasibility studies were excluded.

A detailed search strategy was developed utilizing Boolean Logic (AND, OR, and NOT) and Medical Subject Headings. For example, (“brain natriuretic peptide*” OR “BNP” OR “N terminal probnp” OR “NT-proBNP” OR “n-terminal prohormone brain type natriuretic peptide*” OR “nt pro-bnp” OR “troponin” AND “hypertension” OR “high blood pressure” AND “major adverse cardiac event” OR “MACE” OR “Heart failure” OR “myocardial infarction” OR “atrial fibrillation” OR “stroke” OR “end organ damage” OR “all-cause mortality” OR “ hospitalisation”).

### 2.2. Selection of Studies for Inclusion in the Review

Following the set inclusion and exclusion criteria, three reviewers (EM, KW, and JY) independently screened identified studies by title/abstract, and these were further verified by fourth reviewer (GM). All retrieved studies were exported to EndNote software, version 20 [26] and duplicates removed manually. Rayyan software (https://new.rayyan.ai/, accessed on 20 February 2025) [27] was later utilized to electronically identify and eliminate duplicate results, while any remaining duplicates were manually removed through cross-verification. Full text screening was conducted independently in accordance with the predefined inclusion/exclusion criteria by three reviewers. Conflicts at each stage of the review process were resolved through discussion or adjudication by an independent reviewer. Critical Appraisal Skills Programme (CASP) was used to assess quality and risk of bias.

### 2.3. Data Collection and Management

Our methodology followed the recommendation in the Cochrane Handbook for Systematic Reviews and Interventions [28]. Extracted data included author, year of publication, study design, biomarkers, outcome, sample size, and effect measures. Meta-analysis was not performed due to heterogeneity in the data and variability in thresholds of biomarkers, reported outcomes, and the definitions of MACE.

## 3. Results

### 3.1. Search Strategy

**Figure 1:**
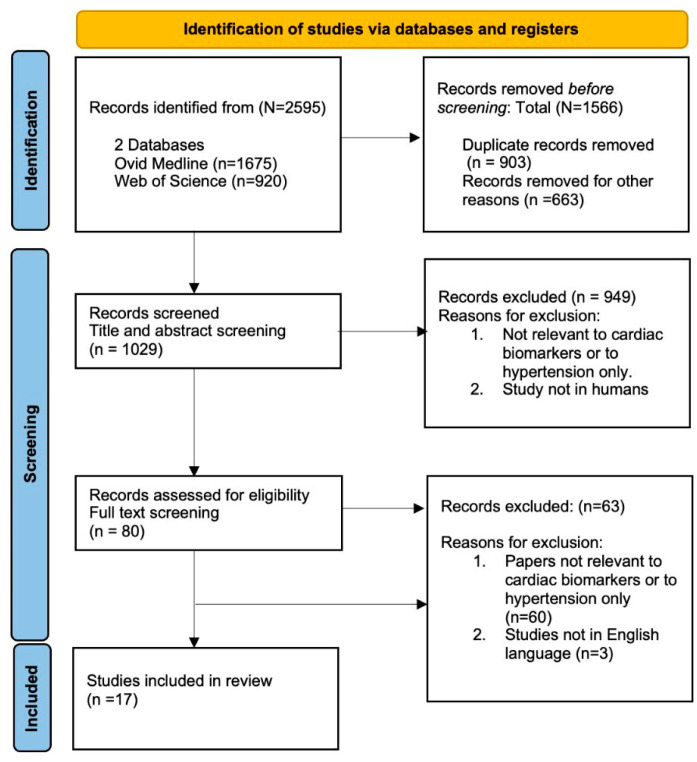
Flow chart showing inclusion and exclusion of studies used according to PRISMA (Preferred Reporting Items for Systematic Reviews and Meta-Analyses) statement.

Our search strategy identified 2595 studies, with 1029 remaining after duplicate removal for title and abstract screening. Following full-text review of 80 articles, 17 studies [29,30,31,32,33,34,35,36,37,38,39,40,41,42,43,44,45,46,47] met the inclusion criteria. The included studies were published between 2013 and 2025. An online search was last conducted on 20 February 2025 (Figure 1).

### 3.2. Quality Appraisal of Included Studies

We assessed the methodological quality of included studies using the CASP checklist [48]. The checklists examined the quality of study types, including cross-sectional, retrospective cohort, and clinical trials. Findings from the CASP assessment were used to categorize studies based on quality, ensuring a transparent and critical evaluation of the included literature (Supplementary Table S1). Studies deemed to have significant methodological limitations or high risk of bias were noted, and their potential impact on the overall findings considered in the interpretation of results. The included papers were independently appraised by two reviewers, with any disagreements resolved through discussion to reach a consensus.

### 3.3. Demographics and Study Design

Seventeen studies, comprising a total of 70,021 participants, were included in this review. Study designs varied, encompassing 8 prospective cohort studies, 6 randomized controlled trials (RCTs), 3 retrospective studies. These studies investigated the prognostic value of BNP (*n* = 6), NT-proBNP (*n* = 9), cTnT (*n* = 4), and cTnI (*n* = 7) in relation to adverse outcomes. Eight studies predicted composite MACE with reported hazard ratios ranging from 1.24 to 4.07. Among the secondary individual outcomes, all-cause mortality (reported in 7 studies; HR range: 1.2–2.6), heart failure (6 studies; HR: 1.4–3.9), and atrial fibrillation (3 studies; HR: 1.0–1.3) were the most frequently reported (Table 2). Cardiac biomarkers showed a strong association with reported outcomes. One study found no significant association between BNP and troponin I levels and the incidence of subclinical atrial fibrillation (AF). Follow-up period varied considerably across studies, ranging from 6 months to 17.3 years (Table 2).

### 3.4. Prognostic Utility of BNP and NTpro BNP

A total of 15 included studies investigated the prognostic significance of BNP (*n* = 6) and NT-proBNP (*n* = 9). The cohort included 59,746 patients, with a mean age of 64.9 (44.3–80) years. The average sex distribution was 51% females. The included papers were limited by significant heterogeneity in the reported outcomes and definition of MACE. BNP and NT-proBNP were significantly correlated with all-cause mortality with HR ranging from 1.9 to 4.18, and HF with HR ranging from 1.6 to 3.8. The inclusion of BNP or NT-proBNP in risk prediction models, included as a continuous or dichotomous variable, further limited any formal statistical synthesis of the data. Further the reported effect size was inconsistent, with some authors reporting various combinations of hazard ratio (HR), odds ratio (OR), and (RR). Notably, one study [44] found no significant association between BNP and troponin I levels and the incidence of subclinical atrial fibrillation (AF) among its 82 patients. A summary of the included studies may be found in Table 2.

### 3.5. Prognostic Utility of Cardiac Troponins

A total of 11 included studies investigated the prognostic significance of high sensitivity cTnT (*n* = 4) and cTnI (*n* = 7). The cohort included 54,010 patients, with a mean age of 67.7 (54.9–80). The average sex distribution was 47.8% females. The included papers were limited by significant heterogeneity in the reported outcomes and definition of MACE. Reported study outcomes included MACE with HR ranging from 1.19 to 4.08. Consistent with studies of the NPs, the inclusion of cardiac troponins in risk prediction models, included as a continuous or dichotomous variable, further limited any formal statistical synthesis of the data. Further the reported effect size was again inconsistent, with some authors reporting various combinations of hazard ratio (HR), odds ratio (OR), and Relative Risk (RR). Cardiac troponins were significantly correlated with all-cause mortality with HR ranging from 1.46 to 2.6. Troponins were significantly correlated with variously defined composite outcomes. A summary of the included studies may be found in Table 2.

## 4. Discussion

The result from this comprehensive systematic review indicates that elevated natriuretic peptides and cardiac troponin T and troponin I in hypertensive patients were associated with an increased risk of MACE and its individual components. This review highlighted that there has been growing evidence on the prognostic utility of these routine biomarkers in patients with hypertension who are at a raised risk of developing HF and other cardiovascular events. Findings from this review showed heterogeneity of the literature in terms of methodology and clinical definition of composite outcomes (MACE).

While current clinical guidelines do not recommend routine circulating biomarkers for CVD risk stratification in patients with hypertension [49], with emerging evidence increasingly supporting their utility in high risk populations, biomarkers are becoming central to efforts aimed at enhancing CVD risk prediction. Their prognostic utility lies in their ability to detect even minor pathophysiological signals incorporating cardiac, vascular, and renal systems [50,51]. These biomarkers provide insight into cardiovascular structure and function, including myocyte injury (cardiac troponin), inflammation, and fibrosis of the heart. Similarly, NPs play a protective role from through their natriuretic, vasorelaxant, metabolic, and antiproliferative systemic properties preventing the progression of HF [52].

In current clinical practice, NPs and troponin biomarkers are currently recommended for risk stratification in chronic HF [53], myocardial infarction (AMI), acute coronary syndromes (ACS), and non-ACS myocardial injury [20,54]. The utility of these biomarkers in HF has resulted in significant improvements in predicting mortality [55]. The elevation of these biomarkers are early signs of heterogenous underlying diseases that are linked to poorer patient outcomes [56,57] with studies indicating its presence in patients with chronic hypertension. This elevation is indicative of subclinical myocardial injury or stress resulting from sustained hypertension, potentially leading to myocardial fibrosis and progressive cardiac dysfunction [58]. However, a rise in cardiac troponin levels can be caused by several other factors that reflect myocyte injury [59]. Contrary to this, elevation of troponin levels has also been identified in healthy individuals [60]. While several pathways of its release remain unclear [61], its elevation has been consistently associated with MACE [56,57].

Risk prediction utilizing cardiac biomarkers has also been demonstrated beyond hypertension in population-based studies. The Framingham Heart Study revealed that minimally elevated troponin I levels in diabetic patients without established cardiovascular disease correlated with increased incident HF risk [62]. The ARIC study demonstrated that elevated hs-cTnT (≥14 ng/L) conferred a 2.5-fold increased risk of incident HF in diabetic patients compared with those without diabetes [63]. Similarly, results from the CANVAS trial found elevated NT proBNP associated with significantly higher HF risk in diabetic patients [64].

Prospective observational studies have also detected circulating biomarkers in general healthy populations. A meta-analysis involving 87,747 participants showed a 3-fold risk for cardiovascular events in subjects with elevated BNP [65]. Several other studies have evaluated the role of NPs and troponin in healthy individuals without prior CVD and its higher risk for mortality [66,67,68,69]. In hypertensive patients without established HF, the prognostic utility of NPs remains insufficiently examined, as noted in this review. Contrary to this, there is a plethora of literature examining the prognostic role of NPs in HF; however, the variability in biomarker thresholds, cost-effectiveness, and lack of standardization across populations and clinical settings continues to pose challenges for their application in risk prediction among hypertensive patients.

The additional combined use of multiple biomarkers with other CVD risk tools could enhance precision of cardiovascular risk stratification in patients with hypertension [70]. However, the potential of multi-biomarkers in risk stratification raises challenges including a lack of consistent evidence in improving cardiovascular outcomes [71], standardized protocols for measurement, interpretation, and clinical application. Moreover, current guidelines [21] remain without any specific thresholds or instructions to use these biomarkers for risk stratification for patients with hypertension. This could be attributed in part due to the heterogeneity in current RCTs. Further investigation into the precise mechanisms by which these biomarkers reflect end-organ damage in hypertension is crucial for advancing their prognostic value.

### 4.1. Limitations

While the findings of this review suggest promise for risk stratification among patients with hypertension, some limitations must be considered. The majority of the included studies had relatively small sample sizes, which may affect the generalizability of their findings. Furthermore, considerable variability existed across studies in terms of design, outcome measures, follow-up periods—from 6 months to 12 years—lack of standard biomarker cutoff values, and number of patients enrolled, which could contribute to heterogeneity. While the majority of the included studies adjusted for key confounders such as age and renal function, the extent and consistency varied with limited details in some studies. This inconsistency may introduce residual confounding in the observed results. The main covariates adjusted for in the analysis of individual studies may be found in Supplementary Table S2.

### 4.2. Clinical Implications

Cardiovascular risk screening remains the cornerstone of primary prevention of cardiovascular diseases. Integrating both established and emerging biomarkers offers a promising approach for improving risk stratification in patients with hypertension, tailoring treatment strategies, and ultimately improving patient care.

### 4.3. Future Perspective

Future research should focus on standardizing thresholds for these biomarkers in risk stratification for hypertension, exploring their role in diverse hypertensive subgroups, and assessing their potential in risk stratification in large scale studies. There is a need for reclassification of the definition of MACE as an outcome [72,73] and further research is warranted to fully understand the cost-effectiveness of adding these biomarkers to existing prediction tools.

Additionally, prospective studies are needed to confirm their utility in improving patient outcomes, particularly in high-risk populations with comorbidities. Most studies included in this review originated from certain regions, highlighting a gap in research representation from other areas implying that these biomarkers may not be widely accessible or integrated into clinical practice in these regions. To address this disparity, policy changes are necessary to promote equitable access and adoption of these biomarkers, ensuring that marginalized and underserved populations, particularly in low- and middle-income countries, can benefit from their clinical utility.

## 5. Conclusions

The prognostic utility of biomarkers in management of hypertension continues to be an area of active research. This systematic review suggests that elevated levels of BNP, NT-proBNP, and cardiac troponins (T/I) are consistently associated with an increased risk of MACE in patients with hypertension without HF. However, due to methodological heterogeneity, variable biomarker thresholds, and limited confounder adjustment across studies, these findings should be interpreted with caution. While these biomarkers have shown promise for enhancing CVD risk stratification, further large-scale prospective studies are warranted to establish their routine clinical utility. Their clinical significance should be a cornerstone for CVD risk stratification.

## Figures and Tables

**Table 1:** Picot framework for the systematic review.

Population	Participants Were > 18 Years, Any Sex and Ethnicity Who Had a Diagnosis of Hypertension or High Blood Pressure. Studies Reporting on Pregnant Women were Excluded.
Exposure	Circulating biomarkers (Natriuretic peptides—BNP/NT-proBNP and cardiac troponin (T or I)). Studies reporting on atrial natriuretic peptides were excluded
Outcomes	End organ damage was the outcome and was defined as a record/diagnosis of one of the following: Primary outcome: Incident major adverse cardiac event (MACE)—defined as a composite outcome of myocardial infarction; heart failure; atrial fibrillation; heart failure hospitalisation; stroke; and all-cause mortality Secondary outcome: Any component of the composite primary outcome
Time frame	Studies included in this narrative review were from 2013 up to February 2025: Ovid Medline and Web of Science
Setting	The study applies to any clinical settings involved in the management of hypertension, including but not limited to primary care, hospital-based care (e.g., emergency departments, cardiology units, general medicine wards, intensive care units, and coronary care units).

**Table 2:** Characteristics of included studies.

Study Title	Country Journal Published	Study Design	Sample Size	Age Range Mean Age, Sex %	Length of Follow Up	Biomarkers Tested Threshold/Cutoff	Clinical Outcomes	Main Results/Findings with HR/OR/with 95% CI
**Ali et al., 2023**	Pakistan Internal and Emergency Medicine	Retrospective study	180	Participants were aged 18 years and above, with a mean age of 54.9 ± 13.4 years. Females accounted for 56.1%	2 years	High sensitivity Troponin I	End organ damage Kidney Heart	Out of 180 patients, 164 (91%) had troponin administered. In total, 34 (18.9%) patients had abnormal values New onset EOD was diagnosed in 15 (8.3%) patients. In total, 73.3% developed EOD in form of kidney followed by (26.6%) in heart (HR 1.27) cTnI Median IQR was 0.012 (0.006–0.054) for end organ damage, significant difference from no end organ damage (*p* = 0.001)
**Ballo et al., 2015**	Italy Clin Res Cardiol	Prospective cohort study	1012	Participants mean age was 66.6 years with males comprising 48.1%	Over median follow up of 49.8 ± 6.7 months	NT-proBNP threshold: 141.4 pg/mL for women (sensitivity 70.0%, specificity 71.3%) and 128.9 pg/mL for men (sensitivity 50.0%, specificity 79.3%)	Cardiovascular mortality Heart failure hospitalization Non-fatal myocardial infarction	NT proBNP correlated with age and was higher in women than men 91.9 (51.2–173.9) vs. 65.7 (35.9–119.9) pg/mL, *p* < 0.0001] In 72 patients, 128 had events (4 cardiac deaths, 45 hospitalizations for HF, and 79 acute coronary syndromes) NT-proBNP plasma concentration as a continuous variable was a predictor of events [HR 2.7 (1.9–3.6), *p* < 0.0001] Elevated NT proBNP analysed as a dichotomous variable was associated with a threefold higher event rate compared with normal levels (RR 3.0, 95% CI 1.9–4.6: *p* < 0.0001)
**Jarret et al., 2023**	U.S.A and Peurto Rico Circulation (AHA)	RCT	9361 patients	Participants were older than 50 years, with a mean age of 68.7 years. The cohort included 35.0% females and 65.0% males	Follow-up period of 1 year	High sensitivity- cardiac troponin T > 6 ng/L NT-proBNP > 125 pg/mL	Primary outcome was composite of all-cause mortality and HF Secondary outcomes included all-cause mortality, the composite CVD end point	Higher hs-cTnT levels were associated with older age, male sex, higher systolic and lower diastolic blood pressure, and impaired renal function, reflected by lower eGFR and elevated urinary albumin. Similarly, elevated NT-proBNP levels were linked to older age, white race, reduced renal function, and lower systolic and diastolic blood pressure. The hazard ratio for the composite outcome of CVD and mortality was 1.85 (95% CI 1.04–3.28). For all-cause mortality alone, the HR was 2.63 (95% CI, 1.11–6.26). The combined outcome of heart failure and all-cause mortality showed an HR of 3.47 (95% CI, 1.54–7.82). For NT-proBNP: The HR for all-cause mortality was 2.40 (95% CI, 1.06–5.43). For the combined outcome of heart failure and all-cause mortality, the HR was 3.72 (95% CI, 1.68–8.23)
**Xiaoming et al., 2024**	U.S.A and Puerto Rico Clinical chemistry.	RCT	8796	Participants were older than 50 years, with a median age of 67 years (IQR: 61–76). The cohort included 3238 females (36.8%) and 5558 males (63.2%)	Follow-up period of 1 year	High-sensitivity cardiac troponin T and I NT proBNP	Heart failure All cause death primary composite CVD (myocardial infarction, other acute coronary syndrome, stroke, HF, and cardiovascular death) ASCVD (myocardial infarction, other acute coronary syndrome, stroke, and cardiovascular death)	Increases in NT-proBNP and hs-cTnT levels from baseline to one year were independently associated with a higher risk of major cardiovascular outcomes. For NT-proBNP, primary composite CVD (HR 1.24, 95% CI: 1.11–1.37; *p* < 0.001), ASCVD (HR 1.16, 95% CI: 1.03–1.30; *p* = 0.02), heart failure (HR 1.66, 95% CI: 1.32–2.08; *p* < 0.001), and all-cause mortality (HR 1.15, 95% CI: 1.00–1.32; *p* = 0.05). Similarly, elevated hs-cTnT composite CVD (HR 1.19, 95% CI: 1.02–1.40; *p* = 0.03), heart failure (HR 1.44, 95% CI: 1.04–1.98; *p* = 0.03), and all-cause death (HR 1.46, 95% CI: 1.18–1.80; *p* < 0.001), though not significantly with ASCVD (HR 1.11, 95% CI: 0.93–1.32; *p* = 0.23)
**Jarett et al., 2021**	U.S.A. JAMA Cardiol.	RCT	9361 patients enrolled in SPRINT	Participants were older than 50 years with a mean age of 68.0 years (SD 9.5); 5915 (63.2%) were male and 3446 (36.8%) were female	Follow-up period of 4 years	The threshold values of NTproBNP were 125 pg/mL or more and hscTnT of 14 ng/L or more	The primary outcome was composite of all-cause mortality and HF Secondary outcomes included all-cause mortality and the composite CVD endpoint	The median hscTnT concentration was 9.4 ng/L, with 25.6% of patients exhibiting levels ≥ 14 ng/L. The median NT-proBNP concentration was 86 pg/mL, with higher values observed in women (112 pg/mL) compared with men (73 pg/mL) In fully adjusted models, elevated hscTnT was associated with a higher risk of both primary and secondary outcomes: -composite of all-cause mortality and HF (421 events; [HR], 1.60 [95% CI, 1.26–2.04]), -all-cause mortality (339 events; HR, 1.56 [95% CI, 1.19–2.05]), -composite CVD end point (531 events; HR, 1.26 [95% CI, 1.02–1.57]), -combined composite CVD end point and all-cause mortality (713 events; HR, 1.39 [95% CI, 1.15–1.67]) Similarly, NT-proBNP levels ≥ 125 pg/mL were linked to greater risk of the all-cause mortality and HF composite (HR 2.26, 95% CI 1.76–2.89), all-cause mortality (HR 1.98, 95% CI 1.51–2.60), composite CVD endpoint (HR 1.81, 95% CI 1.47–2.23), and the combined CVD and mortality endpoint (HR 1.82, 95% CI 1.52–2.19) Participants with both hs-cTnT ≥ 14 ng/L and NT-proBNP ≥ 125 pg/mL had significantly higher risks compared with those with lower biomarker levels: all-cause mortality and HF composite (HR 4.75, 95% CI 3.48–6.81), all-cause mortality (HR 3.78, 95% CI 2.68–5.32), composite CVD endpoint (HR 2.82, 95% CI 2.17–3.68), and the combined CVD plus mortality endpoint (HR 3.04, 95% CI 2.42–3.83).
**Agata et al., 2015**	Poland International Journal of Molecular Sciences	Prospective, observational, cohort study	120	Participants in the non–HF group had a mean age of 61.8 ± 11 years and were 45% male. Those in the HF group were older, with a mean age of 64.5 ± 11 years, and predominantly male (86%)	Follow-up period of 8 years	NT-proBNP	Heart failure	NT-proBNP > 332 pg/mL was a significant predictor of heart failure, with an odds ratio (OR) of 3.08 (95% CI, 1.54–6.14).
**Conti et al., 2014**	Italy Critical Pathways in Cardiology	prospective study	1299	Participants had a mean age of 76 ± 10 years, including 653 females (50.3%) and 646 males (49.7%	Follow-up period of 6 months	Troponin I Cut off/measurement not given	Composite endpoint of ischemic vascular events (stroke, acute coronary syndrome, revascularization, and mortality)	Among 113 patients with elevated troponin I, 15 reached the composite endpoint compared with 43 patients without e-TnI (*p* < 0.001). In the elevated TnI group, outcomes included 3 strokes (5%), 8 cases of coronary heart disease (14%), and 2 deaths (4%). In the non-elevated TnI group, there were 3 strokes (5%), 2 cases of coronary heart disease (4%), and 2 deaths (2%). Both univariate and multivariate analyses identified cTnI as a significant predictor of the primary endpoint, with odds ratios of 4.07 (95% CI, 2.2–7.6; *p* < 0.001) and 3.21 (95% CI, 1.7–6.1; *p* < 0.001), respectively.
**Natalie et al., 2023**	U.S.A. American Journal of Hypertension	Retrospective study	10,382 participants	Participants were aged 20 years and above with a mean age 44.3 years, 52.3% women, and 71.5% non-Hispanic White)	Over a median follow up of 17.3 years	NT-proBNP- threshold ≥ 125 pg/mL,	All-cause mortality and CVD mortality	Elevated NT-proBNP all-cause mortality (HR 2.29, 95% CI 1.79, 2.95) and cardiovascular mortality (HR 3.83, 95% CI 2.34, 6.29)
**Everett et al., 2015**	U.S.A. American Heart Association journals/Circulation	Multinational RCT conducted in 26 countries	12, 956 participants	Participants had a mean age of 65.7 years, with 36.2% females and 63.8% males	2.0 years (quartile1–3 [Q1–Q3], 1.5–2.5 years)	A total of 12,956 samples were analyzed for high-sensitivity cardiac troponin I and 11,057 samples for BNP. The tertile cut points for hsTnI were 3.0 and 4.6 ng/L in men, and 2.6 and 3.9 ng/L in women. For BNP, tertile cut points were 20 and 28.6 ng/L in men, and 20 and 44.4 ng/L in women	Study outcomes were major vascular event composite of nonfatal MI, nonfatal stroke, hospitalization for unstable angina, arterial revascularization, or death and all-cause mortality	hsTnI concentrations in the highest tertile (men ≥ 4.6 ng/L; women ≥ 3.9 ng/L) were associated with a first major cardiovascular event (adjusted hazard ratio [aHR], 2.19; 95% CI, 1.56–3.06; *p* < 0.001). BNP levels in the highest tertile (men ≥ 28.6 ng/L; women ≥ 44.4 ng/L) were also linked to a first cardiovascular event (aHR, 1.94; 95% CI, 1.41–2.68; *p* < 0.001). Baseline cardiac troponin I and BNP were associated with the risk of vascular events and all-cause mortality. hsTnI and all-cause mortality (HR, 2.61; 95% CI, 1.81–3.78; *p* < 0.0001), as well as between the composite of the primary endpoint plus all-cause mortality (HR, 2.42; 95% CI, 1.86–3.15; *p* < 0.0001).
**Josephine et al., 2024**	Netherlands 2024 British Journal of General Practice	Prospective cohort study in 5 Dutch general practices btn 2010–2012 and 2020	530 patients	Participants were aged 60 to 85 years, with a mean age of 70 (±6.5) years. Females accounted for 301 (56.8%) and males 229 (43.2%)	9 years of follow up	BNP levels had a median of 10.0 pmol/L (IQR, 5.7–18.0). Elevated BNP, defined as ≥10 pmol/L, was observed in 257 participants (48.5%)	All-cause mortality (ACM) Cardiovascular events (CVEs) Heart failure	Among 530 participants, 31 (5.8%) developed a coronary event, 44 (8.3%) a cerebrovascular accident, 53 (10.0%) atrial fibrillation, 23 (4.3%) heart failure, and 66 (12.5%) died Elevated BNP increased the risk of ACM, CVEs, and HF independently by 44% HR 1.44 (95% CI, 1.07, 1.94), *p* = −0.017), 45% HR 1.45, (95% CI, 1.15, 1.82), *p* < 0.002), and 288% (HR 3.88, 95% CI, 2.13, 7.10), *p* < 0.001), respectively
**Gallagher et al., 2018**	Ireland American Journal of Hypertension,	Prospective study-Screening to prevent heart failure (STOP-HF) cohort	572 patients	Participants had a mean age of 64.7 years (SD 9.9), with 309 males (54.0%) and 263 females (46.0%	Median follow up 4.0 years	BNP	MACE-I or 2 of (arrhythmia, transient ischemic attack, stroke, myocardial infarction, peripheral or pulmonary thrombosis/embolus, or heart failure) death, MACE + death	Among 572 patients with uncomplicated and complicated hypertension, there were 33 (5.77%) events of MACE plus death. BNP predicted future MACE/death with an odds ratio (OR) of 2.06 (95% CI, 1.50–2.83; *p* < 0.001). There were 16 MACE events (2.80%) and 17 deaths (2.97%), totaling 33 events (5.77%; *p* = 0.011). Among 427/572 uncomplicated hypertension patients, BNP had OR 2.08, 95% CI 1.35, 3.19) *p* < 0.001 of predicting MACE Among 145/572 complicated hypertension patients, BNP had a 1.75 (1.05, 2.91) *p* = 0.032 of predicting MACE
**Giannopoulos et al., 2015**	Greece Journal of Heart Rhythm society	Post hoc analysis of a prospective RCT study	296 patients	Participants were aged between 54 and 66 years, with a mean age of 60 years. There were 207 males (70%) and 89 females (30%)	Over median follow up of 13.7 months	NT-proBNP- cut off point of ≥125 pg/mL was used; however, thresholds of ≥300 pg/mL and ≥450 pg/mL were assessed as well	Atrial Fibrillation (AF recurrence)	NT-proBNP showed a significant univariate correlation with AF recurrence, with each higher quartile of NT-proBNP corresponding to a 47%, 95% CI 21.5–77.9%) (*p* < 0.001) increase in the risk of recurrence All patients with NT-proBNP at baseline btn (155–211–338 pg/mL) had a HR 1.29, 95% CI 0.98–1.68 *p* = 0.66 while patients (*n* = 190) with normal LVEF had 1.31 95% CI 0.96–1.80, *p* = 0.94
**Kim et al., 2022**	Korea Scientific Reports Journal	Retrospective cohort study	3099 patients	Participants were aged over 18 years, with a median age of 68 years (IQR: 53–79). Females comprised 46.3% (*n* = 1435)	Follow-up period of 5.2 years	BNP tertiles were defined as follows: Tertile 1 (BNP ≤ 37 pg/mL), Tertile 2 (BNP > 37 pg/mL and <167 pg/mL), and Tertile 3 (BNP ≥ 167 pg/mL)	Long term mortality	Within a 3-year follow-up period, all-cause mortality occurred in 6.4% of patients in the first tertile of BNP, 24.8% in the second tertile, and 44.4% in the third (highest) tertile Compared with patients in the first tertile of BNP, those in the second tertile had a significantly higher risk of 3-year all-cause mortality (adjusted HR 2.64; 95% CI, 1.96–3.55), as did those in the third tertile (adjusted HR 4.18; 95% CI, 3.09–5.64)
**Okuyama et al., 2017**	Japan Heart and Vessels Journal	Prospective study	493 patients	Participants were older adults with a mean age of 68.5 years (SD ± 10.2), comprising 355 males (72%) and 138 females (28%)	Mean follow up 86.1 months	Troponin I (hs-TnI) levels were stratified into 3 categories: Lowest: <5.0 pg/mL Middle: 5.0–10.6 pg/mL Highest: ≥10.6 pg/mL NT-proBNP levels were categorized as follows: Lowest: <74.1 pg/mL Middle: 74.1–239.7 pg/mL Highest: ≥239.7 pg/mL	Incident heart failure	During a mean follow-up period of 86.1 months, 44 heart failure (HF) admissions were recorded, 31 due to HF with preserved ejection fraction (HFpEF) and 13 due to HF with reduced ejection fraction (HFrEF; LVEF < 50%). Both high-sensitivity troponin I (hsTnI) and NT-proBNP levels were found to be independent predictors of HF admission, whether analyzed as continuous or categorical variables: as continuous variables: hsTnI: HR 2.56 (95% CI, 1.31–5.00; *p* = 0.005) NT-proBNP: HR 3.55 (95% CI, 1.82–6.91; *p* = 0.0002), as categorical variables (≥highest tertile): hsTnI: HR 3.10 (95% CI, 1.53–6.28; *p* = 0.002) NT-proBNP: HR 3.17 (95% CI, 1.45–6.90; *p* = 0.004) The combined elevation of both hsTnI and NT-proBNP was strongly associated with an increased risk of HFpEF admission, with a hazard ratio of 9.45 (95% CI, 2.47–35.4) when compared with participants with neither biomarker elevated
**Philippsen et al., 2022**	Denmark Pacing and Clin Electrophysiology Journal	Prospective, single-center observational study	82 Patients	Participants were aged ≥65 years, with a mean age of 71.3 years (IQR: 67.4–75.1). A total of 52 participants (63%) were male	Median follow-up of 588 days (IQR: 453–712 days)	Cardiac troponin I (cTnI) Brain natriuretic peptide (BNP)	Incident subclinical Atrial Fibrillation	After a median follow up of 588 days, 20.7% (17 patients out of 82) developed incident sub clinical atrial fibrillation. Multivariate analysis, both biomarkers, troponin I and BNP, were not associated with incident subclinical AF: (BNP OR 1.00 95% CI 0.99–1.02) cTnI (OR 0.99, 95% CI 0.96–1.05)
**Pokharel et al., 2015**	U.S.A. Hypertension-AHA Journal	Population-based observational study	11,191 participants	Participants had a mean age of 63 years (SD 6), with females comprising 6263 (55.9%) of the cohort	Median follow up of 12 years	Cardiac troponin T (cTnT): Mean level was 7.5 ng/L (SD 17) with cutoff categories defined as <3, 3–5, 6–8, 9–13, and >14 ng/L. NT-proBNP: Median concentration was 68 pg/mL (IQR 33–134)	Incident CV disease (CHD, stroke and HF hospitalization)	Approximately 53% of cardiovascular events occurred in patients with cTnT levels ≥ 3 ng/L Among patients with SBP 140–149 mmHg and cTnT ≥ 14 ng/L, the risks were elevated as follows: incident heart failure hospitalization (HR 4.3; 95% CI, 2.7–6.8; *p* = 0.002), coronary heart disease (HR 2.1; 95% CI, 1.4–3.2; *p* = 0.092), hard CHD (HR 2.0; 95% CI, 1.2–3.3; *p* = 0.006), and stroke (HR 3.0; 95% CI, 1.6–5.6; *p* = 0.173
**Bahr et al., 2024**	Germany European Journal of GP	Explorative sub-analysis of randomised clinical trial- SCREEN-AF	291 patients	Participants were 75 years and above had a mean age of 80 ± 3 years, with 171 females (59%) and 120 males (41%)	Follow-up period up to 6 months	BNP NT-proBNP high sensitivity troponin I	Atrial fibrillation	At 6 months, 8 of 291 patients developed incident atrial fibrillation (AF). Compared with those without AF (*n* = 283), patients with AF (*n* = 8) had significantly higher median levels of BNP [78 (IQR 64.5–112) vs. 41 (27–77) ng/L, *p* = 0.0121], NT-proBNP [273 (201.05–587.1) vs. 186 (111.1–319) ng/L, *p* = 0.0293], and hs-cTnI [7.4 (4.15–16.2) vs. 3.9 (3.1–5.9) ng/L, *p* = 0.0129]

## Data Availability

This systematic review was conducted using publicly available data from previously published studies. All data sources are accessible in the public domain, and no new primary data were generated or collected as part of this study.

## Supplementary Materials

The following supporting information can be downloaded at: https://www.mdpi.com/article/10.3390/jcm14175935/s1, Table S1: (a) showing quality of appraisal of studies using CASP checklist for RCT studies; (b) showing quality of appraisal of studies using CASP checklist for cohort studies. Table S2: variables adjusted for in statistical analysis reported by individual studies including in this review. These are as reported in the individual papers.
