# Supplementary material for: Evaluating the Prognostic Significance of Circulating Biomarkers of End Organ Damage in Hypertension
Source: J Clin Med. 2025 Aug 22;14(17):5935. doi: 10.3390/jcm14175935 (PMC12429671; doi:10.3390/jcm14175935)
Supplement: Supplementary file 1 [file jcm-14-05935-s001.zip › jcm-3778434-supplementary.pdf]

Supplementary Tables

Supplementary Table S1a: showing quality of appraisal of studies using CASP checklist for RCT studies

Study Title	Did the study address a clearly formulated research question?	Was the assignment of participants to interventions randomised?	Were all participants who entered the study accounted for at its conclusion?	(a) Were the participants 'blind' to intervention they were given? (b) Were the investigators 'blind' to the intervention they were giving to participants? (c) Were the people assessing/analysing outcome/s 'blinded'?	Were the study groups similar at the start of the randomized controlled trial?	Apart from the experimental intervention, did each study group receive the same level of care (that is, were they treated equally)?	Were the effects of intervention reported comprehensively?	Was the precision of the estimate of the intervention or treatment effect reported?	Do the benefits of the experimental intervention outweigh the harms and costs?	Can the results be applied to your local population/in your context?	Would the experimental intervention provide greater value to the people in your care than any of the existing interventions?
Jarret et al., 2023	Yes	Yes	Yes	A&B No, yes, for C	Yes	Yes	Yes	Yes	Yes	Yes	Yes
Xiaoming et al., 2024	Yes	Yes	Yes	Can't tell	Yes	Yes	Yes	Yes	Yes	Yes	Yes
Jarett et al., 2021	Yes	Yes	Yes	A&B No, yes, for C	Yes	Yes	Yes	Yes	Yes	Yes	Yes
Everett et al.,2015	Yes	Yes	Yes	Yes	Yes	Yes	Yes	Yes	Yes	Yes	Yes

Bahr et al., 2024	Yes	Yes	Yes	NO	Can't tell	Yes	Yes	Yes	Yes	Can't tell	Yes

Supplementary Table S1b. showing quality of appraisal of studies using CASP checklist for cohort studies

Study Title	Did the study address a clearly focused issue?	Was the cohort recruited in an acceptable way?	Was the exposure accurately measured to minimise bias?	Was the outcome accurately measured to minimise bias?	Have the authors identified all important confounding factors?(b)Have they taken account of the confounding factors in the design and/or analysis?	Was the follow up of subjects complete enough? Was the follow up of subjects long enough?	What are the results of this study?	How precise are the results?	Do you believe the results?	Can the results be applied to the local population?	Do the results of this study fit with other available evidence?	What are the implications of this study for practice?
Ali et al.,2023	Yes	Yes	Yes	Yes	Can’t tell	No	Yes	Can’t tell	Yes	Yes	Yes	Yes
Ballo et al., 2015	Yes	Yes	Yes	Yes	Can’t tell	Yes	Yes	Yes	Yes	Yes	Yes	Yes
Agata et al., 2015	Yes	Can’t tell	Yes	Can’t tell	Can’t tell	No	Yes	Yes	Yes	Yes	Can’t tell	Yes
Conti et al., 2014	Yes	Yes	Yes	Yes	Yes	Yes	Yes	Can’t tell	Yes	Yes	Yes	Yes
Giannopoulos et al.,2015	Yes	Yes	Yes	Yes	Can’t tell	Yes	Yes	Yes	Yes	Yes	Yes	Yes
Natalie et.,2023	Yes	Yes	Yes	Yes	Yes	Yes	Yes	Yes	Yes	Yes	Yes	Yes

Josephine et al., 2024	Yes	Yes	Yes	Yes	Can't tell	Yes	Yes	Yes	Yes	Yes	Yes	Yes
Gallagher et al., 2018	Yes	Yes	Yes	Yes	Can't tell	Yes	Yes	Yes	Yes	Yes	Yes	Yes
Kim et al., 2022	Yes	Yes	Yes	Yes	Yes	Yes	Yes	Yes	Yes	Yes	Yes	Yes
Okuyama et al., 2017	Yes	Yes	Yes	Yes	Yes	Yes	Yes	Yes	Yes	Can't tell	Yes	Yes
Philippsen et al., 2022	Yes	Can't tell	Yes	Yes	Can't tell	Can't tell	Yes	Yes	Yes	Can't tell	Yes	Yes
Pokharel et al., 2015	Yes	Yes	Yes	Yes	Yes	Yes	Yes	Yes	Yes	Yes	Yes	Yes

Supplementary Table S2: Variables adjusted for in statistical analysis reported by individual studies including in this review. These are as reported in the individual papers.

Study Title	Age	Sex	Race or ethnicity	DM	CVD	BMI or weight	Smoker	eGFR	Chol	CV/BP Medication	AF	HF	DBP/ SBP	Baseline Biomarker level	LVEF	Others
Ali et al.,2023	+	+		+	+					+						Cerebrovascular accident, Ischaemic heart disease
Ballo et al., 2015	+	+		+		+	+	+	+	+		+			+	Left Ventricular ejection fraction, Left LV mass, Mitral regurgitation Heart rate, metabolic syndrome
Jarret et al., 2023	+	+	+	+	+	+	+	+					+	+		
Xiaoming et al., 2024	+	+	+	+	+	+	+	+	+	+		+		+	+	Statin medications, clinical site
Jarett et al., 2021	+	+	+	+	+	+	+	+	+	+		+		+	+	Clinical site, baseline BP
Agata et al., 2015	+	+		+		+	+	+		+		+				
Conti et al., 2014	+	+		+		+	+	+		+						
Natalie et.,2023	+	+	+	+	+	+	+	+	+	+		+			+	Education, physical activity

																and alcohol consumption
Everett et al.,2015	+	+	+	+	+	+	+	+	+	+		+			+	Study drug, family history of myocardial infarction<br>High sensitivity C-reactive protein
Josephine et al., 2024	+	+			+	+	+	+	+			+	+	+		Adjusted for Framingham covariates
Gallagher et al., 2018	+	+		+		+	+	+	+			+	+	+		
Giannopoulos et al.,2015	+	+		+		+	+	+	+		+			+	+	Treatment with moxonidine<br>Left atria diameter
Kim et al., 2022	+	+		+	+		+	+	+	+			+			Coronary artery disease (CAD)
Okuyama et al., 2017	+	+		+	+		+	+	+		+		+		+	Haemoglobin, previous myocardial infarction,<br>Follow up duration
Philippsen et al., 2022	+	+		+	+		+	+	+		+		+		+	
Philippsen et al., 2022								+								

Pokharel et al., 2015	+	+		+	+		+	+	+		+		+	+		Fasting glucose, stroke
Bahr et al., 2024																

DM = Diabetes Mellitus; CVD = Cardiovascular Disease; BMI = Body Mass Index; eGFR = estimated Glomerular Filtration Rate; Chol = Total Cholesterol; AF = Atrial Fibrillation; HF = Heart Failure; SDP/DBP = Systolic or Diastolic Blood Pressure; LVEF = Left Ventricular Ejection Fraction.
